# Molecular characteristics of *mcr-1*-carrying plasmids and new *mcr-1* variant recovered from polyclonal clinical *Escherichia coli* from Argentina and Canada

**DOI:** 10.1371/journal.pone.0180347

**Published:** 2017-07-05

**Authors:** Nathalie Tijet, Diego Faccone, Melina Rapoport, Christine Seah, Fernando Pasterán, Paola Ceriana, Ezequiel Albornoz, Alejandra Corso, Alejandro Petroni, Roberto G. Melano

**Affiliations:** 1 Public Health Ontario Laboratory, Toronto, Ontario, Canada; 2 Servicio Antimicrobianos, National and Regional Reference Laboratory in Antimicrobial Resistance, Instituto Nacional de Enfermedades Infecciosas (INEI)-ANLIS “Dr. C. Malbran”, Buenos Aires, Argentina; 3 Consejo Nacional de Investigaciones Científicas y Técnicas (CONICET), Buenos Aires, Argentina; 4 Department of Laboratory Medicine and Pathobiology, University of Toronto, Toronto, Ontario, Canada; Ross University School of Veterinary Medicine, SAINT KITTS AND NEVIS

## Abstract

We have characterized nine *mcr*-*1-*harboring plasmids from clinical *Escherichia coli* isolates previously described in Argentina and Canada. Three of these plasmids carried a *mcr-1*-variant called here *mcr-1*.*5*. All these *E*. *coli* isolates were not clonally related and were recovered in different years and locations. However, their *mcr-1*-harboring plasmids showed high identity among them and to others characterized in other countries, which strongly suggests that this plasmid-type is playing an important role in spreading this mechanism of resistance to polymyxins.

## Introduction

Since the first description of MCR-1, a plasmid-encoded phosphoethanolamine transferase, in November, 2015, in China [1], this mechanism of polymyxin resistance was detected around the world in enterobacterial isolates recovered from animals, environment, food samples and humans [2]. MCR-1-producing *Escherichia coli* was the most common species described in the literature, mainly of polyclonal origin. Similar plasmids harboring *mcr-1* gene (pMCRs) were found elsewhere, belonging to replicon types IncI2, IncHI2 and IncX4, supporting the notion that horizontal transfer constitutes the major dissemination route of *mcr* genes [3,4].

Very recently a new *mcr* allele, *mcr-2* (76.7% nucleotide identity with *mcr-1*), was characterized in Belgium [5], as well as new variants of *mcr-1* (*mcr-1*.*2* in Italy and *mcr-1*.*3* and *-1*.*6* in China) [6–8].

The first gram-negative *mcr-1*-positive bacteria described in the Americas were *E*. *coli* isolates recovered in Argentina [9] and Canada [10]. Nine multidrug resistant *E*. *coli* were recovered from clinical human specimens in six Argentinian hospitals from three cities between July 2012 and January 2016. These isolates were part of a larger sample of 87 colistin resistant Gram-negative bacilli collected between 2008 and 2016, which were screened for the presence of *mcr-1* by PCR [9]. The Canadian isolate was recovered from a gastrostomy tube site and rectum of a patient hospitalized in Ottawa, Ontario, Canada, in 2011, who previously received health care in Egypt [10,11]. This isolate was an OXA-48- and CTX-M-15-producing multidrug-resistant *E*. *coli*. As part of a database of ~1600 Canadian bacterial whole-genome sequences, this was the only clinical isolate of three *mcr-1*-positive *E*. *coli* initially found in Canada after screening of this database [10]. Here, we describe the molecular characteristics of IncI2 pMCRs recovered from these clinical *E*. *coli* isolates from Argentina and Canada, as well as a new variant of the *mcr-1* gene. We also compare them with other IncI2 *mcr*-harboring plasmids described elsewhere, strengthening the notion of this replicon-type as one of the main *mcr*-disseminator.

## Materials and methods

The first ten *mcr-1* isolates described in the Americas were included in this study, nine from Argentina (*E*. *coli* isolates M15049, M15224, M17056, M17059, M19241, M19242, M19441, M19736 and M19855) and one from Canada (*E*. *coli* GN775) [9–11]. Susceptibility profiles were obtained by Etest (bioMérieux) with the exception of colistin (broth dilution) and the results interpreted by the 2016 Clinical and Laboratory Standards Institute guidelines [12] except for colistin and tigecycline, interpreted according to the European Committee on Antimicrobial Susceptibility Testing guidelines [13]. *E*. *coli* clinical isolates were genotyped by MLST [14]. Plasmid profiles of these clinical isolates were obtained by pulsed-field gel electrophoresis analysis of S1 nuclease-digested DNA (S1-PFGE) [15]. The ones carrying the *mcr-1* gene were identified and their sizes estimated by S1-PFGE followed by Southern blot analysis using a specific *mcr-1* probe [15]. Plasmid content of each isolate was extracted with the QIAprep Spin miniprep kit (Qiagen) and used for transformation assays in chemically competent *E*. *coli* TOP10 (Life Technologies; colistin MIC of 0.016 μg/ml). *mcr-1*-transformant strains were selected using Luria-Bertani agar plates supplemented with colistin (1 μg/ml), and confirmed by PCR. pMCRs were then extracted from transformant *E*. *coli* strains using the Qiagen Large-Construct kit (Qiagen) and sequenced using Illumina’s MiSeq system. The obtained contigs were assembled using CLC Genomics Workbench software (CLC bio, Qiagen). Gaps were filled by PCR amplification and Sanger sequencing. Open reading frames (ORFs) were annotated using the RAST server (rast.nmpdr.org) followed by manual comparative curation and determination of sequence similarity using the BLAST web server. Alignments with other IncI2 pMCRs were performed by using the BRIG tool [16].

The sequences of the plasmids reported here have been deposited in GenBank under accession numbers KY471307 (pMCR-GN775), KY471308 (pMCR-M15049), KY471309 (pMCR-M15224), KY471310 (pMCR-M17059), KY471311 (pMCR-M19241), KY471312 (pMCR-M19242), KY471313 (pMCR-M19441), KY471314 (pMCR-M19736) and KY471315 (pMCR-M19855)

## Results and discussion

Table 1 shows the susceptibility profiles of *E*. *coli* clinical isolates. Colistin MICs ranged from 4 to 16 μg/ml. All isolates were susceptible to amikacin and tigecycline. With the exception of the Canadian isolate (OXA-48 and CTX-M producer) [11] and 4 Argentinian *E*. *coli* (CTX-M producers) [9], the isolates were generally susceptible to ß-lactams. Conversely, most of the isolates were resistant to quinolones and tetracycline. By MLST all the clinical isolates belonged to different sequence types (ST) (Table 1). Compared to the *E*. *coli* MLST database (http://mlst.ucc.ie/mlst/dbs/Ecoli; 7,412 STs; last accessed June 5, 2017), none of the STs assigned to the Argentinian isolates included in this work were found among the eight entries previously reported from Argentina, while ST624, which was assigned to the Canadian isolate GN775, was found in two entries reported from Canada. ST410, which was assigned to isolate M19441, was defined as a hyperepidemic clone and founder of the widely disseminated clonal complex 23 (CC23) [17]. This clone was also previously found to carry *mcr-1* as well as *bla*_CTX-M_ genes in isolates recovered from a turkey hen meat sample in Germany [18] and from a human blood culture in Brazil [19]. In these two last cases, *mcr-1* gene was located on the bacterial chromosome [18] and on an IncX4 plasmid [19]. There is evidence that *E*. *coli* ST410 has been successful for interspecies transmission between food-producing animals, wildlife, humans, companion animals and the environment, increasing the risk of becoming a successful pandemic clone [20].

**Table 1:** Antimicrobial susceptibility profiles and sequence types (ST) of clinical *E*. *coli* isolates.

Isolates^a^	MIC (μg/ml)^b^																			MLST (ST)^c^
	AMP	FOX	CAZ	CTX	FEP	IPM	EPM	MEM	AZM	AKN	GEN	TOB	NAL	CIP	SXT	TET	TGC	FOF	COL	
**GN775**	≥256	16	12	≥256	16	1	6	1	24	2	64	6	≥256	≥32	≥32	≥256	0.094	256	4	624
**M15049**	3	2	0.75	0.047	0.032	0.25	0.003	0.016	0.064	2	1	0.75	≥256	≥32	≥32	64	0.5	128	8	6756
**M15224**	≥256	8	1	2	0.75	0.125	0.004	0.008	0.19	4	1	1	≥256	16	≥32	64	0.25	≥1024	16	641
**M17059**	≥256	4	0.75	128	3	0.19	0.008	0.023	0.75	2	32	6	≥256	≥32	0.032	64	0.19	1	8	1488
**M19241**	3	4	0.5	0.094	0.047	0.19	0.003	0.012	0.064	4	1.5	1	≥256	≥32	0.047	64	0.38	1.5	8	1196
**M19242**	≥256	6	4	≥256	32	0.5	0.023	0.023	16	3	4	8	2	0.016	≥32	48	0.38	2	4	1049
**M19441**	≥256	12	128	≥256	128	0.38	0.064	0.023	≥256	8	≥256	48	≥256	≥32	0.38	128	0.125	2	8	410
**M19736**	≥256	3	0.094	0.032	0.047	0.19	0.003	0.16	0.032	1.5	0.25	0.25	≥256	≥256	0.064	48	0.38	6	4	615
**M19855**	≥256	6	32	96	16	0.19	0.064	0.023	32	6	192	96	≥256	32	0.19	≥256	0.125	8	8	602

^**a**^ *E*. *coli* M17056, one of the nine Argentinian clinical isolates described in ref. 9, was not included in this table because no *mcr-1*-transformant strain was achieved.

^**b**^ AMP, ampicillin; FOX, cefoxitin; CAZ, ceftazidime; CTX, cefotaxime; FEP, cefepime; IPM, imipenem; EPM, ertapenem; MEM, meropenem; AZM, aztreonam; AKN, amikacin; GEN, gentamicin; TOB, tobramycin; NAL, nalidixic acid; CIP, ciprofloxacin; SXT, trimethoprim-sulfamethoxazole; TET, tetracycline; TGC, tigecycline; FOF, fosfomycin; COL, colistin. Results were interpreted according to Clinical and Laboratory Standards Institute guidelines [12], except for colistin and tigecycline, for which the European Committee on Antimicrobial Susceptibility Testing breakpoints were used [13].

^c^ MLST, Multilocus Sequence Typing. The allelic numbers and STs were assigned online using: http://mlst.ucc.ie/mlst/dbs/Ecoli

**Fig 1:**
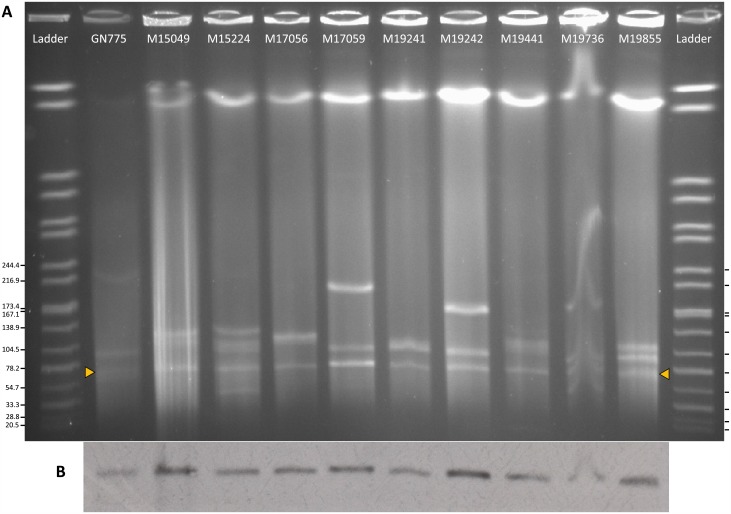
Identification of *mcr*-1-harboring plasmids. (A) S1 nuclease-pulsed–field gel electrophoresis plasmid profiles. (B) Autoradiograph of gel A hybridized with *mcr*-1 probe. Yellow arrowheads indicate positive bands. Ladder, reference standard *Salmonella enterica* serotype Braenderup strain H9812 restricted with *Xba*I (sizes are given in kilobases).

S1-PFGE showed a varied plasmid content in all the clinical isolates but Southern blot with *mcr-1* probe showed that the pMCRs were very similar in size (~60 kb) (Fig 1).

Nine *mcr-1*-transformant strains were obtained (8 from Argentina and 1 from Canada; no *mcr-1* transformant strain was achieved for *E*. *coli* M17056). Colistin was the only antimicrobial drug for which all transformant isolates showed reduced susceptibility or resistance (MICs of 2 to 4 μg/ml). Comparison of the transferred *mcr* genes with the original *mcr-1* [1] showed that 3 of the Argentinian isolates (M15049, M17059 and M19241, recovered from two hospitals) had the same missense point mutation in position 1,354 resulting in a H452Y change of the MCR protein. Other three new MCR-1 variants were recently published, MCR-1.2, MCR-1.3, and MCR-1.6 [6–8]. Other unpublished MCR-1 variants were also found in the Genbank database (Table 2). We noticed that one of them, called MCR-1.5 found in an *E*. *coli* isolated from human urinary tract sample in Argentina (GenBank accession number KY283125), had the same H452Y amino acid change described in our study. To avoid future confusions with the nomenclature, we called the new variant described here as MCR-1.5.

**Table 2:** *mcr-1* variants available at the GenBank database (updated to June 9, 2017).

Variant	Amino acid change	Species	Country	Accession number	Reference
*mcr-1*	-	*E*. *coli*	China	KP347127	[1]
*mcr-1*.*2*	Q3L	*K*. *pneumoniae*	Italy	KX236309	[6]
*mcr-1*.*3*	I38V	*E*. *coli*	China	KU934208	[7]
*mcr-1*.*4*	D440N	*E*. *coli*	China	KY041856	Unpublished
*mcr-1*.*5*	H452Y	*E*. *coli*	Argentina	KY283125KY471308KY471310KY471311	Unpublished This work
*mcr-1*.*6*	R536H	*S*. Typhimurium	China	KY352406	[8]
*mcr-1*.*7*	A215T	*E*. *coli*	China	KY488488	Unpublished
*mcr-1*.*8*	Q3R	*E*. *coli*	Brunei	KY683842	Unpublished

**Fig 2:**
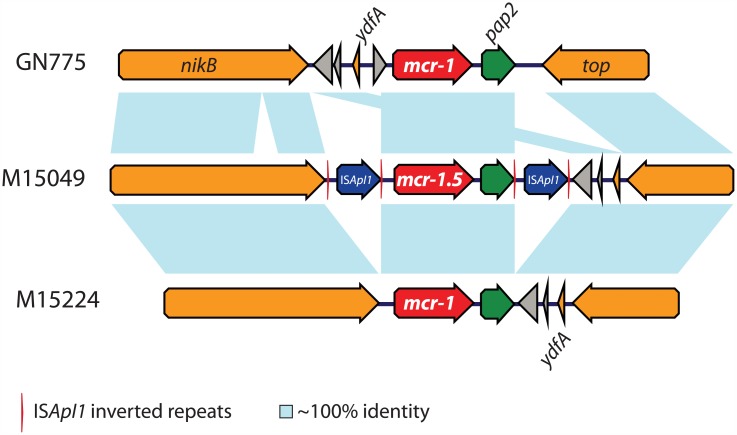
Genetic environment of *mcr-1* variants. Genes and their corresponding transcriptional orientations are indicated by horizontal broad arrows. Open reading frames encoding hypothetical proteins are represented by grey triangles. Vertical red lines represent IS*ApI1* inverted repeats (IRL and IRR).

All plasmids included the conserved 2,607 bp DNA segment containing *mcr* (*mcr-1* or *mcr-1*.*5*) and *pap2* genes [21] with slightly different genetic environments (Fig 2). Two isolates presented the change TAAAAT instead of TATAAT at -10 box promoter region described previously [22]. The three isolates carrying the new variant *mcr-1*.*5/pap2* (M15049, M17059 and M19241) had this DNA segment flanked by 2 copies of IS*ApI1* in the same orientation (Fig 2) but the characteristic 2 bp target site duplications (TSD) were not found [21].

This composite transposon was not described very often (usually only one copy, if any, of IS*ApI1* is present beside *mcr-1*) [4,20,23] and would be responsible for the chromosomal integration of *mcr-1* in *E*. *coli* [18,23–25]. IS*ApI1* was not present in pMCRs from the other 5 Argentinian and the Canadian isolates (Fig 2). The corresponding inverted repeat right and left (IRR and IRL) were also absent in those plasmids. However, the *mcr-pap2* element was found in a similar location, between *top* (encoding a DNA topoisomerase III) and *nikB* (relaxase) genes (Fig 2), like in pHNSHP45 and other IncI2 plasmids, suggesting a hot spot for the insertion of this transposon in this plasmid-type. In these 6 isolates where IS*ApI1* was absent, the *mcr-pap2* element was flanked by a conserved trinucleotide (5’-ATA-3’), a sequence found immediately downstream of the IRR of IS*Apl1* in both composite transposons and single-copy IS*Apl1* [21]. The absence of IS*ApI1* in some pMCRs described here and elsewhere could be explained by its mobilization, transposing the *mcr-pap2* structure to conjugative plasmids with a subsequent loss of IS*ApI1* copies following integration, as postulated before [21,26,27].

**Fig 3:**
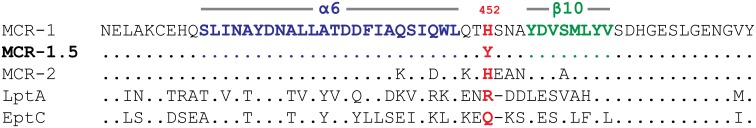
Alignment of the deduced amino acid sequence of MCR-1 and MCR-1.5 with other related phosphoethanolamine transferases. Position 452 (His in MCR-1; Tyr in MCR-1.5) is indicated in red between helix α6 (blue) and sheet β10 (green).

Based on the predicted structure of MCR-1 [1,28] and the crystal structure of the catalytic domain [29], the amino acid change H452Y found in MCR-1.5 was located between helix α6 and sheet β10 (Fig 3).

Like MCR-1, a H452 was found in MCR-2, which showed some amino acid changes surrounding this position. Homology comparison with the phosphoethanolamine transferases (PAE) LptA from *Neisseria meningitidis* and EptC from *Campylobacter jejuni* showed an arginine (position 440) and glutamine (position 415), respectively, corresponding to the Y452 in MCR-1.5. A tyrosine residue was also observed at this position in the PAE of *Enhydrobacter aerosaccus* (WP_007116571.1), *Paenibacillus sophorae* (WP_036596266.1) and *Dichelobacter nodosus* (WP_041729850.1) [1]. A recent evolutionary analysis grouped MCR-1 with those of *E*. *aerosaccus*, *P*. *sophorae* and *D*. *nodosus*, and very close to the PAE of *Moraxella catarrhalis* (subclade I), while LptA was grouped in a second subclade of PAEs [30]. The lack of differences in colistin resistance levels observed between MCR-1- and MCR-1.5-transformant strains would suggest that the amino acid change found in MCR-1.5 is not affecting its PAE activity.

**Fig 4:**
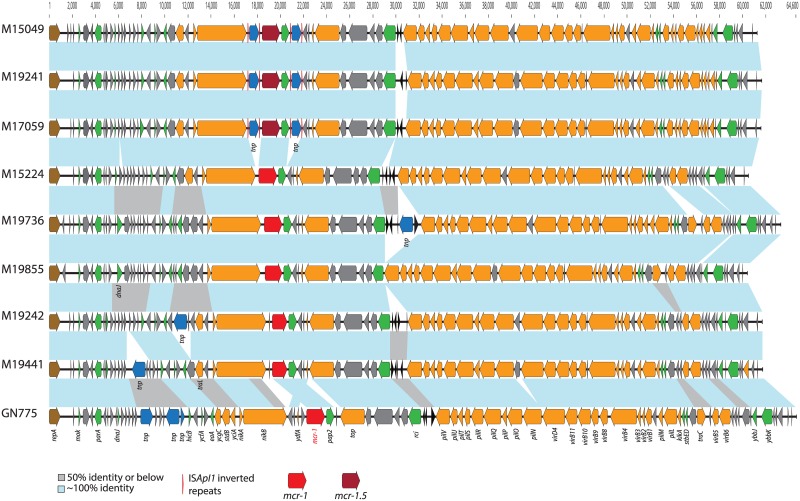
Comparison of pMCRs described in this study. Arrows indicate the following predicted open reading frames: conjugation, stability, and accessory genes (green, yellow), antimicrobial resistance genes (*mcr-1* in red; *mcr-1*.*5* in dark red), transposon-related genes (blue), hypothetical proteins (grey), shufflon segments (black), and replicase genes (brown). The light blue-shaded areas show regions with ~100% identity among the compared structures; grey-shaded areas, 50% identity or lower.

Full sequences of these nine pMCR showed a GC content ranging between 42.3% and 43%. As showed by S1-PFGE and Southern blot analysis, their sizes were similar and all belonged to the IncI2 incompatibility group (Fig 4).

**Fig 5:**
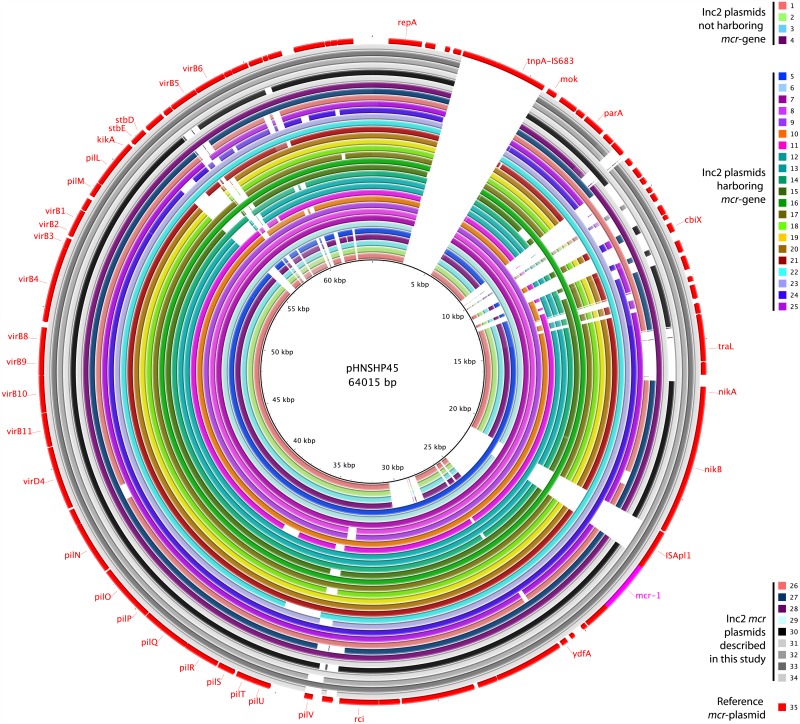
Sequence alignment of IncI2-type *mcr-1*-bearing plasmids. pHNSHP45 was used as a reference to compare with the pMCRs described here and with other IncI2 plasmids. The outer circle with red arrows indicates annotation of the reference sequence. Gaps in the inner circles are missing regions when compared with the reference. Plasmids characteristics are included in Table 3.

pMCRs of Argentinian origin were highly similar, with a genetic identity ranging between 87.2% and 99.8%. Six of them (from clinical isolates M15049, M15224, M17059, M19241, M19242, and M19441) showed maximum genetic similarities to plasmids pABC149-mcr-1 (GenBank accession no. KX013538), isolated from a pathogenic human *E*. *coli* in the United Arab Emirates [31], and pHNSHP45 (KP347127) the first reported pMCR [1]. The other 2 pMCRs (from M19736 and M19855) were more similar to plasmid pECJS-61-63 (KX084393) from an *E*. *coli* recovered in China (95.1% identity; 84.6% identity with pHNSHP45). The Canadian pMCR (from GN775) was the most divergent, with an overall 83% identity with the Argentinian plasmids, 80.3% identity with pHNSHP45, and a maximum genetic identity with plasmid pECJS-61-63 (91%). All backbone and transfer genes in the pMCRs characterized in this study were highly conserved. Differences were mainly due to the presence of insertion sequences (e.g. IS*ApI1* in three Argentinian plasmids flanking the *mcr*-*pap2* structure, or some transposases downstream of *repA* in the Canadian pMCR), absence of open reading frames encoding for hypothetical proteins and reorganization of the *pilV* shufflon (Fig 4). Same kind of similarities and variations were observed in the alignment of different IncI2 plasmids, harboring or not *mcr-1* gene, with the ones described in our study (Fig 5).

**Table 3:** List of IncI2 plasmids aligned in Fig 5 against the pMCRs described in this study.

Fig 5 #	Plasmid name	Species	Length (bp)	Accession #	Country	*mcr*-variant
1	pHNY2	*Escherichia coli*	65358	KF601686	China	No
2	pHNLDH19	*Escherichia coli*	62194	KM207012	China	No
3	pHN1122-1	*Escherichia coli*	62196	JN797501	China	No
4	pSTH21	*Salmonella enterica*	62139	LN623683	Hong Kong	No
5	pA31-12	*Escherichia coli*	67134	KX034083	China	*mcr-1*
6	pEZE36	*Escherichia coli*	65846	KY802014	China	*mcr-1*
7	pMRY16-002_4	*Escherichia coli*	61805	AP017614	Japan	*mcr-1*
8	pMRY15-131_2	*Escherichia coli*	60722	AP017622	Japan	*mcr-1*
9	pMRY15-117_2	*Escherichia coli*	61223	AP017619	Japan	*mcr-1*
10	pWF-5-19C_mcr-1	*Cronobacter sakazakii*	65203	KX505142	China	*mcr-1*
11	pVT553	*Escherichia coli*	62219	KU870627	South Africa	*mcr-1*
12	pSCS23	*Salmonella enterica*	65419	KU934209	China	*mcr-1*
13	pS2.14–2	*Escherichia coli*	60950	CP016187	Malaysia	*mcr-1*
14	pmcr1_IncI2	*Escherichia coli*	64964	KU761326	China	*mcr-1*
15	pMCR_1410	*Kluyvera ascorbata*	57059	KU922754	China	*mcr-1*
16	pEG430-1	*Shigella sonnei*	61826	LT174530	UK	*mcr-1*
17	pECJS-61-63	*Escherichia coli*	63656	KX084393	Hong Kong	*mcr-1*
18	pEC13-1	*Escherichia coli*	60218	CP016186	Malaysia	*mcr-1*
19	pEC5-1	*Escherichia coli*	61735	CP016185	Malaysia	*mcr-1*
20	pBA77-MCR-1	*Escherichia coli*	62661	KX013539	Bahrain	*mcr-1*
21	pBA76-MCR-1	*Escherichia coli*	64942	KX013540	Bahrain	*mcr-1*
22	pAF23	*Escherichia coli*	61177	KX032519	South Africa	*mcr-1*
23	pABC149-MCR-1	*Escherichia coli*	61228	KX013538	UAE	*mcr-1*
24	pA31-12	*Escherichia coli*	67134	KX034083	China	*mcr-1*
25	pmcr1_IncI2	*Escherichia coli*	64964	KU761326	China	*mcr-1*
26	pGN775	*Escherichia coli*	64600	KY471307	Canada	*mcr-1*
27	pM19855	*Escherichia coli*	60357	KY471315	Argentina	*mcr-1*
28	pM19441	*Escherichia coli*	61653	KY471313	Argentina	*mcr-1*
29	pM19242	*Escherichia coli*	61632	KY471312	Argentina	*mcr-1*
30	pM19736	*Escherichia coli*	63230	KY471314	Argentina	*mcr-1*
31	pM15224	*Escherichia coli*	60735	KY471309	Argentina	*mcr-1*
32	pM17059	*Escherichia coli*	61531	KY471310	Argentina	*mcr-1*.*5*
33	pM19241	*Escherichia coli*	61584	KY471311	Argentina	*mcr-1*.*5*
34	pM15049	*Escherichia coli*	61198	KY471308	Argentina	*mcr-1*.*5*
35	pHNSHP45	*Escherichia coli*	64015	KP347127	China	*mcr-1*

In Argentina, *mcr* spread would be due to the increased use of polymyxins against emergent, highly resistant clinical pathogens such as KPC-producing *Enterobacteriaceae* or extremely drug-resistant *Acinetobacter baumannii* [9], which could create selective pressure in Argentinian nosocomial environments. Data from the WHONET-Argentina Resistance Surveillance Network showed that, in 2015, the prevalence of nosocomial *E*. *coli* and *K*. *pneumoniae* non-susceptible to colistin (resistance plus intermediate categories) was 2.2% (1.1% for each intermediate resistance and resistance) and 10.3% (0.2% for intermediate resistance and 10.1 for resistance), respectively [32]. However, a recent report describes a broad dissemination of *mcr-1* gene in *E*. *coli* recovered from healthy poultry in Argentina, suggesting an important source of this colistin resistant microorganism since at least 2013 [33]. In contrast, in countries like Canada where colistin is not commonly used in clinical therapy schemes, low detection of this mechanism in clinical isolates [34] was the result of ‘imported’ pathogens by patients hospitalized in countries where the *mcr* gene is more common to be found (e.g. China) [35], or where it was detected but its prevalence is unknown (e.g. Egypt) [11,36].

The worldwide emergence of colistin resistant bacteria without any prior clinical colistin exposure could be considered a consequence of polymyxins use in veterinary medicine [37–39] and as promoter factors in the agriculture and food production sectors [1,33,37–40]. Abundant bibliography supports this presumption: *mcr*-carrying *Enterobacteriaceae* are frequently isolated from different animal species such as cattle, chicken, and pigs [4,41]. *Moraxella* spp., which includes different species that are mainly animal pathogens, were identified very recently as potential natural reservoir of chromosomal *mcr*-like genes, mobilizing them to *Enterobacteriaceae* [42]. Detection of *mcr-1* also in river water, vegetables and wild birds [43–45] should impulse more collaborative studies with a One Health perspective to bring light to this complex matter. In addition, strong restrictions to the use of antimicrobials in the agriculture sector have to be implemented, particularly for the ones that are still useful drugs for human disease treatments (such as polymyxins).

In summary, we have characterized pMCRs from the first *mcr-1* positive *Enterobacteriaceae* described in the Americas, detected in Argentina and Canada. All these clinical *E*. *coli* isolates were not clonally related and were recovered in different years and locations. However, their pMCRs had high identity among them and other IncI2 pMCRs characterized in other countries, which strongly suggest that this plasmid-type is playing an important role in spreading this mechanism of resistance to polymyxins.
